# PreserFlo Microshunt for the management of intraocular pressure elevation in iridocorneal endothelial syndrome

**DOI:** 10.1016/j.ajoc.2023.101932

**Published:** 2023-09-22

**Authors:** Sunil Ruparelia, Rami Darwich, Brennan D. Eadie

**Affiliations:** a Faculty of Medicine, Dalhousie University, Halifax, Nova Scotia, Canada; b Department of Ophthalmology and Visual Sciences, Dalhousie University, Halifax, Nova Scotia, Canada

**Keywords:** PreserFlo Microshunt, ICE syndrome

## Abstract

**Purpose:**

To report on a case of angle-closure glaucoma secondary to iridocorneal endothelial (ICE) syndrome effectively managed with the PreserFlo Microshunt.

**Observations:**

We report successful implantation of a PreserFlo Microshunt in a 57-year-old patient with secondary angle-closure glaucoma in the context of ICE syndrome. Following failure of medical therapy to adequately control intraocular pressure (IOP), the patient was consented for surgical intervention and underwent combined cataract surgery and PreserFlo Microshunt implantation. IOP at the last post-operative follow-up (5 months) was 12 mmHg with the patient on brinzolamide/timolol maleate (Azarga®). We report no complications in the post-operative period.

**Conclusions and importance:**

The PreserFlo Microshunt may be a promising option for patients with ICE syndrome who fail medical therapy. Implantation of this device was well tolerated in the presented case.

## 1 Introduction

Iridocorneal Endothelial Syndrome (ICE) syndrome is a group of diseases characterized by endothelial metaplastic transformation into “epithelial-like” cells.^1^ These altered corneal endothelial cells may migrate in a membrane-like form and spread onto the iridocorneal angle and iris, leading to peripheral anterior synechiae (PAS) and iris anomalies such as atrophy, correctopia, and polycoria.^2^^,^^3^ Migration of corneal endothelium and the formation of PAS can result in obstruction of the angle and subsequently elevated intraocular pressure (IOP). This can be associated with corneal edema and secondary angle-closure glaucoma.

Although a viral etiology has been proposed, the precise etiology of ICE syndrome remains unknown.^4^ Therapy is directed towards managing the sequalae of the disease, including IOP elevation in an effort to slow the progression of glaucoma and control corneal edema. While the first line treatment is medical therapy, this is frequently ineffective in adequately lowering IOP in ICE syndrome.^5^ A large proportion of patients with ICE syndrome will therefore require surgical intervention. Various IOP-lowering surgical options are available, including trabeculectomy. However, studies evaluating the outcomes of trabeculectomy in patients with ICE syndrome have reported only moderate surgical success and repeat trabeculectomy in these patients is associated with a high rate of failure.^6^^,^^7^

The relatively new PreserFlo Microshunt (Santen, Osaka, Japan) is an 8.5 mm long subconjunctival stent with a 70 μm lumen, implanted in an *ab externo* fashion.^8^ Similar to the XEN Gel Stent (Allergan Inc., Dublin, Ireland), this creates an alternative pathway for aqueous humor from the anterior chamber to the subconjunctival space.^9^ In its early use, the PreserFlo Microshunt has proven effective in managing IOP elevation in various glaucoma subtypes.^10^ We report on the successful use of the PreserFlo Microshunt in a 57-year-old male presenting with secondary angle-closure glaucoma in the context of ICE syndrome.

## 2 Case report

A 57-year-old male was referred to a tertiary glaucoma clinic with reports of elevated IOP in the context of a recent diagnosis of ICE syndrome in his right eye. Ten years prior to his diagnosis with ICE syndrome, he had presented to the emergency eye clinic for right eye irritation and conjunctival injection. At that time, he was diagnosed with right eye non-granulomatous anterior uveitis with increased IOP (30 mmHg) and was found to have inferior iris atrophy in the context of a few years’ history of inferior iris heterochromia. Viral swabs were negative for herpes simplex virus (HSV) and varicella zoster virus (VZV). It was not until 10 years after this initial presentation that ICE syndrome was diagnosed.

Ten years later at the time of ICE syndrome diagnosis, presenting symptoms were right eye discomfort, decreased vision, and inferior iris heterochromia. Best corrected visual acuity (BCVA) was 20/40 in the right eye and 20/20 in the left eye. Right eye intraocular pressure was 35 mmHg by Goldmann applanation tonometry. Slit lamp examination at this time showed signs of epithelial and stromal edema in the mid and peripheral right cornea. The patient also had a large inferior iris defect along with displacement of the pupil superiorly. Gonioscopy revealed a closed angle in the right eye. The angle in the left eye was open. The patient was started on brinzolamide/timolol maleate (Azarga®) and latanoprostene bunod ophthalmic solution (Vyzulta®) and was then sent to our glaucoma service for consideration for surgical intervention.

**Fig. 1:**
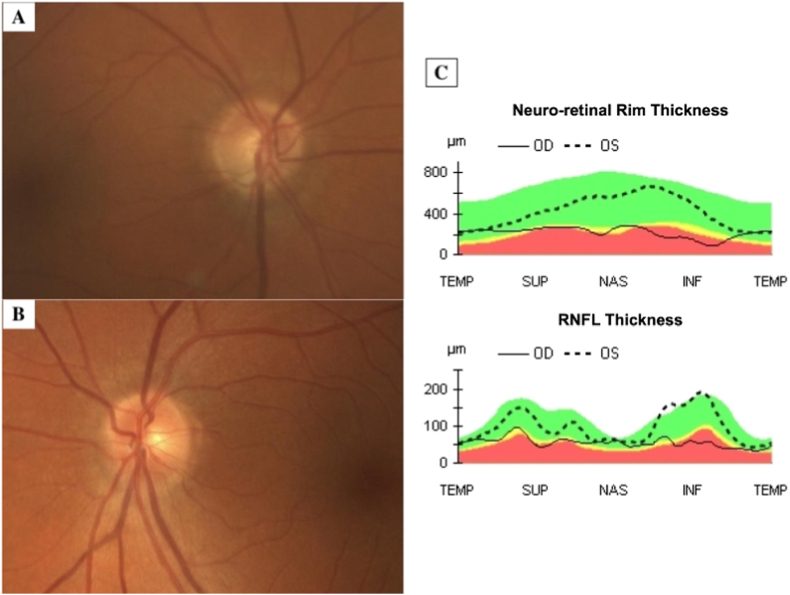
Posterior fundus photography of the right eye (A) and left eye (B) at presentation. Posterior fundus photography shows increased cupping of the right eye. TSNIT plots (C) demonstrate inferior thinning of the optic nerve in the right eye with marked loss of RNFL fibers in the superior and inferior sectors.

**Fig. 2:**
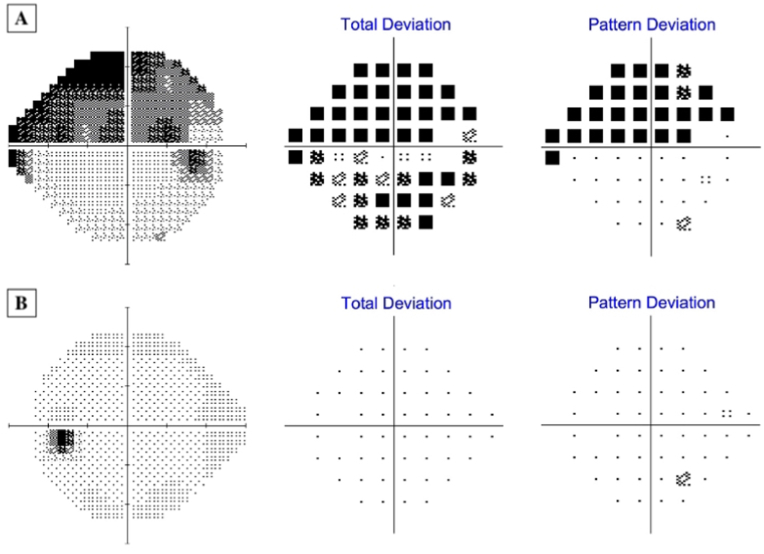
Humphrey Visual Field SITA 24-2 at presentation to glaucoma clinic of the right eye (A) and left eye (B). Note the superior arcuate visual field defect in the right eye. The right eye had a mean deviation of −12.85 dB and a glaucoma hemifield test outside normal limits. The left eye had a mean deviation of 0.77 dB and a glaucoma hemifield test within normal limits.

On examination by our glaucoma service, Snellen BCVA was 20/40 in the right eye and 20/20 in the left eye. Goldman applanation IOP was 22 mmHg in the right eye despite use of three classes of glaucoma medications. Early nuclear sclerotic and lenticular changes were also noted in the right lens. Examination of the right optic nerve showed a cup-disk ratio of 0.6 with a thin neuro-retinal rim inferiorly. Left eye examination was unremarkable with a cup-disk ratio of 0.3. Right eye optic disc optical coherence topography (OCT) demonstrated thinning of both the superior and inferior retinal nerve fibre layer (Fig. 1). Humphrey visual field (SITA 24-2) showed corresponding superior foci of decreased sensitivity, including within the central 10° (Fig. 2). Left eye disc and macula OCT and visual field analysis were within normal limits.

Given the persistent elevation of right eye IOP despite medical therapy in the context of advanced glaucomatous optic neuropathy, the patient was consented for surgical intervention. The patient underwent combined cataract surgery and PreserFlo Microshunt implantation. This procedure was performed in an operating room setting using sterile technique. Phacoemulsification was completed in the standard fashion. Tenon's and conjunctiva were then dissected from sclera and Xylocaine 2% with epinephrine was injected under the Tenon's capsule. Three sponges soaked with 0.4 mg/mL mitomycin C were applied inside the conjunctival/Tenon's pocket for a total of 120 seconds. The sponges were removed, and the conjunctiva washed with 60 cc of saline. A 1-mm slit angled knife at 20° angle was then used to create a shallow scleral pocket. From this scleral pocket, a 25-gauge needle was used to enter the anterior chamber. The PreserFlo MicroShunt was placed at the 12 o'clock position. Tenon's capsule and conjunctiva were closed with 9-0 Vicryl sutures. Moxifloxacin was injected into the anterior chamber. The patient was instructed to take the following post-operative drop regimen: Prednisolone acetate 1% every 2 hours while awake, moxifloxacin 4 times per day, and atropine sulfate 1% every 12 hours for the first 2 weeks followed by a tapering schedule of prednisolone 1% drops.

**Fig. 3:**
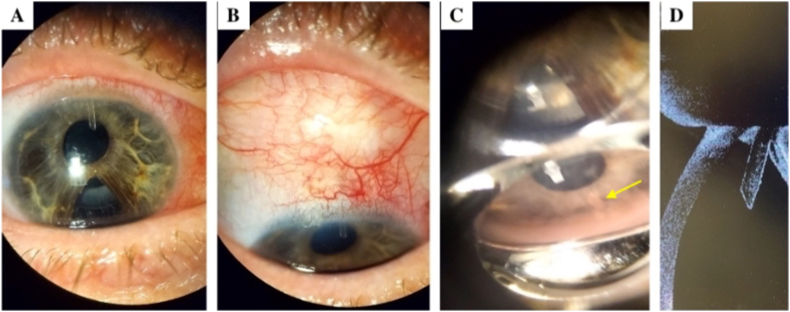
Postoperative Examination of PreserFlo Microshunt Implant in the Right Eye. A) Anterior segment photograph showing the Preserflo MicroShunt in the anterior chamber. B) Photograph of the superior conjunctiva showing a formed filtering bleb. C) Gonioscopy of the superior angle revealing the Preserflo MicroShunt piercing the trabecular meshwork (arrow). D) Anterior segment-OCT (AS-OCT) showing the successfully implanted PreserFlo MicroShunt, positioned bevel up in the anterior chamber.

IOP-lowering drops were discontinued immediately following shunt implantation. Pressure in the right eye at post-operative day 1 was 5 mmHg with an elevated filtering bleb on examination. Pressure remained stable at 5 mmHg during the first post-operative week. In the initial 3 months following PreserFlo Microshunt implantation, IOP ranged from 9 to 12 mmHg off all IOP-lowering medication. At the third post-operative month, IOP elevation to 17 mmHg in the right eye was noted and the patient was restarted on brinzolamide/timolol maleate (Azarga®). IOP was subsequently reduced and remained stable at 12 mmHg up to the last post-operative visit at 5 months. The shunt remained well-positioned with a filtering bleb present throughout the post-operative period (Fig. 3). Snellen BCVA at the last post-operative follow-up was 20/30 in the right eye and 20/20 in the left eye. No complications were reported in the post-operative period to date.

## 3 Discussion

In this single case study, we demonstrate that the PreserFlo Microshunt can be safe and effective in managing uncontrolled IOP in secondary angle-closure glaucoma related to ICE syndrome.

For patients with ICE syndrome, medical therapy alone is frequently ineffective in adequately lowering IOP, and glaucoma filtering surgery has been associated with high rates of failure. A retrospective review of 203 ICE syndrome patients at a tertiary care centre in India showed that despite medical therapy to lower IOP, at least 50% of ICE syndrome patients required surgical intervention.^5^ The success of primary trabeculectomy with MMC in their series was 55% at 5 years.^6^ Of the eyes that underwent surgical intervention, 27% required repeat surgery to control IOP and refractory cases underwent Ahmed glaucoma valve implantation.

Although the literature on use of the PreserFlo Microshunt in ICE syndrome is sparse, several studies have previously evaluated the efficacy of the XEN Gel Stent in managing IOP elevation in ICE syndrome. In a series of 4 cases, Lin et al. demonstrated successful use of the XEN Gel Stent in this context, with no further surgical interventions required in their 7-month follow-up.^11^ A single case study by Hohberger et al. reported successful implantation of XEN Gel Stent in a patient with ICE syndrome after Descemet's membrane endothelial keratoplasty (DMEK) transplantation, further demonstrating the versatility of these devices in complex glaucoma cases.^12^

In the current literature evaluating the PreserFlo Microshunt, early complications have included transient hypotony and transient choroidal effusion, both of which usually resolve spontaneously. Keratitis, hyphema, and bleb fibrosis have also been reported more rarely.^13^^,^^14^ Of note, the PreserFlo Microshunt is primarily indicated for open-angle glaucoma, and therefore implantation in the context of angle-closure glaucoma is off-label. In the present case, PreserFlo Microshunt implantation was well-tolerated with no complications reported in 5-months follow-up to date. A factor to be kept in mind with *ab externo* shunt implantation in ICE syndrome is the position of PAS, which must be avoided to prevent bleeding and maintain a patent device lumen. In *ab externo* implantation, PAS may be harder to visualize than in an *ab interno* approach, and one may need to carefully choose the implant location of the device in order to avoid these synechia. While no repositioning was required in the present case, surgeons may wish to keep this in mind when performing *ab externo* device implantation in the context of ICE syndrome.

## 4 Conclusions

We report on the use of the PreserFlo Microshunt in a case of a 57-year-old male with secondary angle-closure glaucoma related to ICE syndrome. The PreserFlo Microshunt may prove to be an effective treatment option for patients with ICE syndrome who fail medical therapy. Future, larger studies evaluating the PreserFlo Microshunt in the context of ICE syndrome would be valuable.

## Patient consent

Patient written consent was obtained for the preparation of this work.

## Funding

The authors received no financial support for the research, authorship, or publication of this report.

## Authorship

All authors attest that they meet the current ICMJE criteria for Authorship.

## Declaration of competing interest

The authors declare that they have no known competing financial interests or personal relationships that could have appeared to influence the work reported in this paper.
